# Study on the Properties and Synergistic Antioxidant Effects of Novel Bifunctional Fusion Proteins Expressed Using the UTuT6 System

**DOI:** 10.3390/antiox12091766

**Published:** 2023-09-14

**Authors:** Qi Yan, Jingyan Wei, Junxia Song, Mengna Li, Xin Guan, Jian Song

**Affiliations:** 1 College of Pharmaceutical Science, Jilin University, Changchun 130021, China; qiyan20@mails.jlu.edu.cn (Q.Y.);; 2 Key Laboratory for Molecular Enzymology and Engineering of the Ministry of Education, Jilin University, Changchun 130000, China; 3 Institute of Theoretical Chemistry, Jilin University, Changchun 130023, China; 4 School of Microelectronics, Shanghai University, Shanghai 201800, China

**Keywords:** fusion protein, glutathione peroxidase, superoxide dismutase, antioxidant, synergism

## Abstract

Important antioxidant enzymes, glutathione peroxidase (GPx) and superoxide dismutase (SOD), are involved in maintaining redox balance. They can protect each other and result in more efficiently removing excessive reactive oxygen species (ROS), protecting cells against injury, and maintaining the normal metabolism of ROS. In this study, *human cytosolic* GPx (hGPx1) and *human phospholipid hydroperoxide* GPx (hGPx4) genes were integrated into the same open reading frame with *human extracellular* SOD active site (SOD3-72P) genes, respectively, and several novel fusion proteins were obtained by using the UTuT6 expression system for the first time. Among them, Se-hGPx1_UAG_-L_4_-SOD3-72P is the bifunctional fusion protein with the highest GPx activity and the best anti-hydrogen peroxide inactivation ability thus far. The *Se-hGPx4_UAG_-L_3_-SOD3-72P* fusion protein exhibits the strongest alkali and high temperature resistance and a greater protective effect against lipoprotein peroxidation damage. *Se-hGPx1_UAG_-L_4_-SOD3-72P* and *Se-hGPx4_UAG_-L_3_-SOD3-72P* fusion proteins both have good synergistic and antioxidant abilities in H_2_O_2_-induced RBCs and liver damage models. We believe that this research will help with the development of novel bifunctional fusion proteins and the investigation of the synergistic and catalytic mechanisms of GPx and SOD, which are important in creating novel protein therapeutics.

## 1. Introduction

During the processes of aerobic respiration and substrate oxidation in the body, oxygen-containing active substances such as superoxide radicals (O_2_^·−^) and hydrogen peroxide (H_2_O_2_) are produced and constitute reactive oxygen species (ROS) [1]. Excess ROS can induce irreversible oxidative damage to lipids, DNA, and proteins, eventually leading to various diseases [2]. Therefore, it is crucial to maintain the balance of ROS for proper cellular function. The antioxidant defense system, which consists of enzymes and non-enzymes, is the key for maintaining redox balance in living organisms.

As one of the most important antioxidant enzymes, glutathione peroxidase (GPx) has always been a research hotspot. Eight GPx members have been identified. Among them, the active centers of GPx1–4 and GPx6 are selenocysteine (Sec), while GPx5 and GPx7–8 are Cys [3]. GPx1 is widely distributed and highly concentrated, and it also exhibits the strongest activity [3]. It can reduce H_2_O_2_ with GSH as a substrate and can also reduce some low-molecular weight complexes of water-soluble lipid hydroperoxides, for instance, tert-butyl hydroperoxide (t-BuOOH) [4]. GPx4, the only selenoprotein existing as a monomer, specifically reduces complex lipid hydroperoxides like phospholipid hydroperoxides. Ferroptosis, a novel iron-dependent cell death mechanism, has recently attracted significant attention [5], and it is characterized by the accumulation of iron-dependent lipid peroxidation at a lethal level. GPx4 can inhibit some lipid peroxidation, leading to it being considered an important regulator of ferroptosis [6]. In addition, GPx4 has also gained interest in cancer, cardiovascular disease, and neuroscience over the past decades.

The idiosyncratic translational mechanism of Sec makes expressing selenoproteins in vitro challenging [7,8]. In the previous study, based on the Dieter Söll group at Yale University [9], our group developed a method for efficiently expressing GPx with an improved chimeric tRNA (tRNA^UTuT6^) in amber-less *E. coli* [10]. Here, it is named the UTuT6 expression system.

The first and most vital line of defense against ROS is the metalloenzyme superoxide dismutase (SOD) [11]. Three SODs (SOD1–3) have been found in mammals. They all catalyze the dismutation of O_2_^·−^ into oxygen and H_2_O_2_, despite being situated in various locations within cells. Study revealed that SOD3 is expressed in the stratum corneum and close to the connective tissue matrix in the dermis, which was hypothesized to have a direct protective effect on skin inflammation. Compared with SOD1 and SOD2, SOD3 appears to be more involved in the control of immune responses and signal transduction process [12]. Furthermore, SOD3 can regulate the production and migration of dendritic cells (DC), Langerhans cells (LC), and T cells in contact hypersensitivity. Therefore, the expression of a protein with SOD3 activity may play a role in promoting the development of treatments and prevention strategies for skin inflammation.

We were earlier unable to express SOD3 as a soluble protein using BL21 (DE3). SOD3 is the only glycosylated protein in the superoxide dismutase family; however, the prokaryotic expression system lacks post-translational modification and cannot generate glycosylated SOD3, which may be one of the reasons for the inability to obtain soluble SOD3 protein in vitro [13]. Even if SOD3 was subsequently connected to GPx1, expressed as a soluble protein in prokaryotes, it did not improve its solubility. It was discovered that the expression of the SOD3 active site alone might produce soluble protein with SOD activity [14].

In this study, based on the unique role of SOD3, we linked hGPx1, hGPx4, and SOD3 active sites with different lengths of flexible peptides (Linker: (GGGGS)_3_; Long linker: S(GGGGS)_4_), and expressed them in the UTuT6 expression system to obtain bifunctional fusion proteins with both GPx and SOD activities. Among them, Se-hGPx1_UAG_-L_4_-SOD3-72P fusion protein has the highest GPx activity; *Se-hGPx4UAG-L3-SOD3-72P* fusion protein exhibits the strongest alkali and high temperature resistance, and a better protection against lipoprotein oxidative damage. The fusion proteins have similar quaternary structure and catalytic mechanism to the natural GPx and excellent synergistic antioxidant ability in in vitro antioxidant models.

## 2. Materials and Methods

### 2.1. Materials

*E. coli* strain DH5α was used for plasmid propagation. *E. coli* strain C321.ΔA.exp [15] purchased from Addgene (49018), which was grown in the Luria–Bertani medium (LB medium) for protein expression. As a gift from Christopher Voigt (Addgene plasmid #49990, Watertown, MA, USA) [16], plasmid pN565 was transformed into *E. coli* C321.ΔA.exp for T7 RNA polymerase expression under Isopropyl β-D-1-thiogalactopyranoside (IPTG) induction. The plasmids pACYC-[*E. coli selA* + *M. jannaschii pstk*] (pACYC-*selA*/*pstk*) and pGFIB-*tRNA^UTu^* were generous gifts from Dieter Söll [9]. Plasmid pGFIB-*tRNA^UTuT6^* was obtained from a previous study [10] based on pGFIB-*tRNA^UTu^*. NADPH, GSH, IPTG, and glutathione reductase (GR) were obtained from Sigma, St. Louis, MO, USA. Total Sulfhydryl Group Content Assay Kit was procured from Boxbio (Beijing, China). All other chemicals were bought from Beijing Chemical Factory (Beijing, China) and were of analytical grade.

### 2.2. Construction of Fusion Proteins and SOD3-72P Expression Plasmids

The primers *hGPx1_UAG_*-BamHI-F/*hGPx1_UAG_*-linker-R and *hGPx1_UAG_*-BamHI-F/*hGPx1_UAG_*-long linker-R were used to amplify the genes *hGPx1_UAG_*-linker and *hGPx1_UAG_*-long linker from a previous study of ours [10]. Linker-SOD3-72P was amplified using Linker-*SOD3-72P*-F and *SOD3-72P*-HindIII-R. Genes were fused into *hGPx1_UAG_-L_3_-SOD3-72P* and *hGPx1_UAG_-L_4_-SOD3-72P* by a 15-mer peptide linker (GGGGS)_3_ and a 21-mer peptide long linker S(GGGGS)_4_, respectively. After digestion with BamHⅠ and HindIII, the PCR products were ligated into the pRSFDuet-1 expression vector, which had previously been linearized with similar enzymes, and then pRSF-*hGPx1_UAG_-L_3_-SOD3-72P* and pRSF-*hGPx1_UAG_-L_4_-SOD3-72P* were obtained. Construction of pRSF-*hGPx4_UAG_-L_3_-SOD3-72P* and pRSF-*hGPx4_UAG_-L_4_-SOD3-72P* was carried out as outlined for hGPx1_UAG_ fusion proteins, except that *hGPx4_UAG_-L_3_-SOD3-72P* and *hGPx4_UAG_-L_4_-SOD3-72P* were ligated into the EcoRI and HindIII sites of pRSFDuet-1. DNA sequencing (GENEWIZ, Suzhou, China) verified the plasmids. Primer sequences used in this study were listed in Supplementary Table S1, and gene accession numbers used in this study are as follows: GPx1: NM_000581 and GPx4: NM_001367832.

*SOD3-72P* was amplified using *SOD3-72P*-BamHI-F and *SOD3-72P*-HindIII-R. After digestion with BamHⅠ and HindIII, the PCR product was ligated into the pColdI expression vector, which had previously been linearized with similar enzymes, and pColdI-*SOD3-72P* was obtained. DNA sequencing (GENEWIZ, Suzhou, China) verified the plasmids. Primer sequences used in this study were listed in Supplementary Table S1, and gene accession number used in this study is as follows: SOD3: NM_003102.

pRSF-*hGPx4_UAG_-L_3_-SOD3-72P* was used as a PCR template for mutating the Cys codon TGC at 2/10/37/66/75/107 and 148 of hGPx4 to the Ser codon TCG by site-directed mutagenesis, finally resulting in pRSF-*hGPx4_Cys2/10/37/66/75/107/148Ser_-L_3_-SOD3-72P.* DNA sequencing (GENEWIZ, Suzhou, China) verified the plasmids. Primer sequences used in this study were listed in Supplementary Table S2.

### 2.3. Protein Expression and Purification

The bifunctional fusion proteins were obtained according to the method described previously [10]. Recombinant plasmids were transformed into *E. coli* strain C321.ΔA.exp, which had been co-transformed with the plasmids pN565, pGFIB-*tRNA^UTuT6^*, and pACYC-*selA/pstk*. Starting from a single colony after transformation, cells were grown in 1 mL LB medium supplemented with 100 μg/mL of ampicillin, 34.4 μg/mL of chloramphenicol, 50 μg/mL of kanamycin, and 90 μg/mL of spectinomycin at 37 °C. Then, 200 μL overnight culture was added to 200 mL LB medium supplemented with the same resistance, with 50 μM of sodium selenite. When the apparent absorbance of the culture at 600 nm reached 1.2 after incubation at 37 °C for several hours, the temperature was reduced to 20 °C. After 4 h, protein expression was induced with 100 μM IPTG, followed by continued incubation for 18 h at 20 °C. The SOD3-72P protein was expressed according to the method described previously [14].

Cells were harvested using centrifugation at 5000× *g* for 5 min. The expression vectors contain 6× His tag, so an immobilized metal-affinity chromatography purification system with standard Ni^2+^-charged beads was used to purify the fusion proteins after lysis by sonication. The target proteins were eluted with buffer A (50 mM sodium phosphate, 300 mM NaCl, and 300 mM imidazole).

### 2.4. Incorporation of Copper and Zinc into Fusion Proteins

Incorporation of copper and zinc into the fusion proteins was performed by dialysis with 100 µM Cu^2+^ and 100 µM Zn^2+^ at 4 °C for 24 h. The protein concentration was determined with the Bradford method, using bovine serum albumin as the standard.

### 2.5. SDS-PAGE and Western Blot Analysis

After boiling in SDS loading buffer (10 mM Tris-HCl, 4% SDS, 5% β-mercaptoethanol (β-ME), 15% glycerol, and 0.002% bromophenol blue at pH 6.8) for 10 min, purified fusion proteins were subjected to SDS-PAGE followed by Coomassie Brilliant Blue R-250 staining. Non-reduced samples were treated with modified SDS loading buffer (10 mM Tris-HCl, 4% SDS, 15% glycerol, and 0.002% bromophenol blue at pH 6.8) and loaded without boiling.

Starting with SDS-PAGE, samples were then electro-transferred to nitrocellulose membranes. The membranes were blocked with Tween-Tris-buffered saline (TTBS: 0.2% Tween, 100 mM Tris (pH 7.5), and 150 mM NaCl) with 5% nonfat dry milk. Mouse monoclonal anti-His6 IgG was used as a primary antibody. The membranes were washed three times with TTBS, followed by incubation with goat monoclonal anti-mouse IgG conjugated with horseradish peroxidase. Protein bands were visualized using 3,3′-diaminobenzidine (DAB)-H_2_O_2_ staining.

### 2.6. Assay of Enzyme Activities

GPx activity of fusion proteins was assayed according to the method described previously [17]. Samples were mixed with 50 mM PBS (pH 7.4), 1 mM EDTA, 0.25 mM NADPH, and 1 U GR, and incubated at 37 °C for 3 min. The reaction was initiated by addition of 500 µM H_2_O_2_. Activity units (U) were defined as the amount of enzyme utilizing 1 µmol of NADPH per minute. SOD activity was determined using the method described previously [18]. Samples were mixed with 50 mM Tris-HCl (pH 7.4), 0.25 mM EDTA, 1.7 mM Xanthine, and 0.41 mM NBT, and incubated at 37 °C for 3 min. The reaction was initiated by addition of 0.02 U XOD. One unit of SOD activity was defined as the amount of enzyme inhibiting 50% of the rate of NBT reduction. The specific activity was expressed in U/mg. All assays were repeated at least three times.

### 2.7. Molecular Modeling and Docking

The fusion proteins were modeled using Phyre2 [19], and the putative protein structures were further refined by the atomic-level fragment-guided molecular dynamic [20] simulations. GSH was docked within each predicted mutant model using EDock [21].

### 2.8. Effect of Temperature, pH, and Storage Time on Fusion Proteins

The optimal temperatures of *Se-hGPx1_UAG_-L_4_-SOD3-72P* and *Se-hGPx4UAG-L3-SOD3-72P* were assayed in a range from 15 °C to 45 °C at pH 7.4. Similarly, catalytic reactions were carried out from 3 to 11 to determine the optimal pH. The effect of storage time on GPx activity was studied. Purified *Se-hGPx1_UAG_*, *Se-hGPx1_UAG_-L_4_-SOD3-72P*, *Se-hGPx4_UAG_*, and *Se-hGPx4UAG-L3-SOD3-72P* were incubated in 50 mM Tris-HCl at 4 °C, and the activity was measured over a series of time points.

### 2.9. Steady State Kinetics of Fusion Proteins

The kinetics assay of *Se-hGPx1_UAG_-L_4_-SOD3-72P* and *Se-hGPx4UAG-L3-SOD3-72P* was carried out using the method described previously [10]. The steady state parameters were measured with varying concentrations of tBuOOH between 50 and 300 μM while GSH was kept at 1 mM, 2 mM, 3 mM, and 5 mM, respectively, and the activity was measured using the method described above.

### 2.10. Resistance to Inactivation by H_2_O_2_

The cooperation of GPx and SOD activities was detected with *SOD3-72P*, *Se-hGPx1_UAG_-L_4_-SOD3-72P*, and *Se-hGPx4UAG-L3-SOD3-72P* enzymes, which were incubated with 1 mM H_2_O_2_ in 50 mM Tris-HCl (pH 7.4) for various times at 37 °C, and the SOD activity was assayed using the xanthine/XOD system.

### 2.11. Hemolysis Assay

Blood samples were collected from mice with Alsever’s Solution, washed with 0.9% saline (1:3), and centrifuged at 2500 rpm for 10 min at 4 °C to obtain RBCs, then resuspended in 0.9% saline (1% *v*/*v*). The incubation mixture consisted of 0.05 mM GSH, 1% RBCs, appropriate enzymes, and 100 mM H_2_O_2_. Damage experiments were carried out without enzymes; control experiments were performed without enzymes and H_2_O_2_; toxicity control experiments were carried out only with fusion proteins. After treatment for 1 h at 37 °C, the samples were centrifuged at 2500 rpm for 10 min at 4 °C, and the absorbance was detected at 412 nm [22].

### 2.12. Inhibition of Lipid Peroxidation in Liver

The liver was collected from mice removing fat and connective tissue. By adding 20 mL of pre-cooled 0.9% saline per 1 g of liver, liver homogenate with a defined concentration (5% *m*/*v*) was created by using a homogenizer. Protein amount was determined with Coomassie Brilliant Blue using BSA as the standard [23]. The incubation mixture consisted of 0.05 mM GSH, 5% liver homogenate, appropriate enzymes, and 100 mM H_2_O_2_. Damage experiments were carried out without enzymes; control experiments were performed without enzymes and H_2_O_2_. After treatment for 1 h at 37 °C, the absorbance of the MDA-TBA complex was determined at 532 nm using the TBA assay.

### 2.13. Inhibition of Yolk Lipoprotein Peroxidation Induced by FeSO_4_

Yolk suspension was prepared with phosphate-buffered saline (PBS) (1:25 (*m*/*m*)). The incubation mixture consisted of 0.05 mM GSH, 2% yolk suspension, appropriate enzymes, and 25 mM FeSO_4_. After treatment for 1 h at 37 °C, the absorbance of the MDA-TBA complex was determined at 532 nm using the TBA assay.

## 3. Results

### 3.1. Preparation and Identification of Fusion Proteins

**Figure 1:**
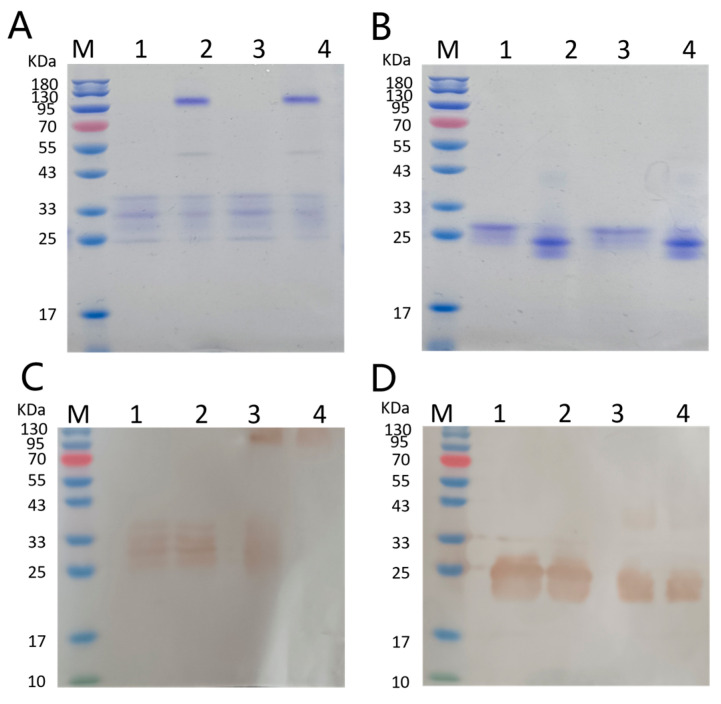
SDS-PAGE and Western blot analysis of the fusion proteins. (**A**) M: Marker; Lane 1: reduced *Se-hGPx1_UAG_-L_3_-SOD3-72P*; Lane 2: non-reduced *Se-hGPx1_UAG_-L_3_-SOD3-72P*; Lane 3: reduced *Se-hGPx1_UAG_-L_4_-SOD3-72P*; Lane 4: non-reduced Se-hGPx1_UAG_-L_4_-SOD3-72P. (**B**) M: Marker; Lane 1: reduced *Se-hGPx4UAG-L3-SOD3-72P*; Lane 2: non-reduced *Se-hGPx4_UAG_-L_3_- SOD3-72P*; Lane 3: reduced *Se-hGPx4UAG-L4-SOD3-72P*; Lane 4: non-reduced *Se-hGPx4_UAG_-L_4_- SOD3-72P*. (**C**) M: Marker; Lane 1: reduced Se-hGPx1_UAG_-L_3_-SOD3-72P; Lane 2: reduced *Se-hGPx1_UAG_-L_4_-SOD3-72P*; Lane 3: non-reduced Se-hGPx1_UAG_-L_3_-SOD3-72P; Lane 4: non-reduced *Se-hGPx1_UAG_-L_4_-SOD3-72P*. (**D**) M: Marker; Lane 1: reduced *Se-hGPx4UAG-L3-SOD3-72P*; Lane 2: reduced *Se-hGPx4UAG-L4-SOD3-72P* Lane 3: non-reduced *Se-hGPx4UAG-L3-SOD3-72P*; Lane 4: non-reduced *Se-hGPx4UAG-L4-SOD3-72P*.

The fusion proteins were identified with reduced and non-reduced SDS-PAGE. The molecular weights of *Se-hGPx1_UAG_-L_3_-SOD3-72P/Se-hGPx1_UAG_-L_4_-SOD3-72P* and *Se-hGPx4UAG-L3-SOD3-72P*/*Se-hGPx4UAG-L4-SOD3-72P* were approximately 32.1 kDa/32.5 kDa and 31 kDa/31.4 kDa, respectively. As shown in Figure 1A,B, reduced selenium containing GPx1 and GPx4 fusion proteins both migrated between 25 and 33 kDa, while non-reduced samples of *Se-hGPx1_UAG_-L_3_-SOD3-72P* and *Se-hGPx1_UAG_-L_4_-SOD3-72P* migrated as two bands composed of reduced and non-reduced fusion proteins of approximately 33 and 130 kDa, and a faint band appeared at approximately 55 kDa, which is not completely reduced. Non-reduced SDS-PAGE analysis of *Se-hGPx4UAG-L3-SOD3-72P* and *Se-hGPx4UAG-L4-SOD3-72P* showed that the samples migrated as a single band of approximately reduced bands, demonstrating that the recombinant fusion proteins were successfully expressed in the UTuT6 expression system and that the quaternary structure of the fusion proteins was similar to that of natural GPx1 and GPx4. Western blot further verified our conclusion (Figure 1C,D). As control, we expressed *SOD3-72P*, whose molecular weight is about 10 kDa (Figure S1).

### 3.2. The Assay of Enzyme Activities

The enzyme activities are shown in Table 1. The GPx activity of *Se-hGPx1_UAG_-L_3_-SOD3-72P* was 190 ± 29 U/mg for the reduction of H_2_O_2_, and the SOD activity was 1537 ± 211 U/mg. When the peptide linker was increased to 21 aa, the GPx activity was 199 ± 16 U/mg, similar to *Se-hGPx1_UAG_-L_3_-SOD3-72P*; however, the SOD activity was increased to 2790 ± 125 U/mg. Unlike GPx1 fusion proteins, the activities of *Se-hGPx4UAG-L3-SOD3-72P* (GPx: 20.78 ± 3.1 U/mg; SOD: 2506.3 ± 223 U/mg) and *Se-hGPx4UAG-L4-SOD3-72P* (GPx: 19.2 ± 2.9 U/mg; SOD: 2403.6 ± 145 U/mg) showed no significant difference. Therefore, we selected *Se-hGPx1_UAG_-L_4_-SOD3-72P* with higher SOD activity and *Se-hGPx4UAG-L3-SOD3-72P* with lower molecular weight to further study their enzymatic properties.

### 3.3. Effect of Temperature, pH, and Storage Time on Se-hGPx1_UAG_-L_4_-SOD3-72P and Se-hGPx4UAG-L3-SOD3-72P

**Figure 2:**
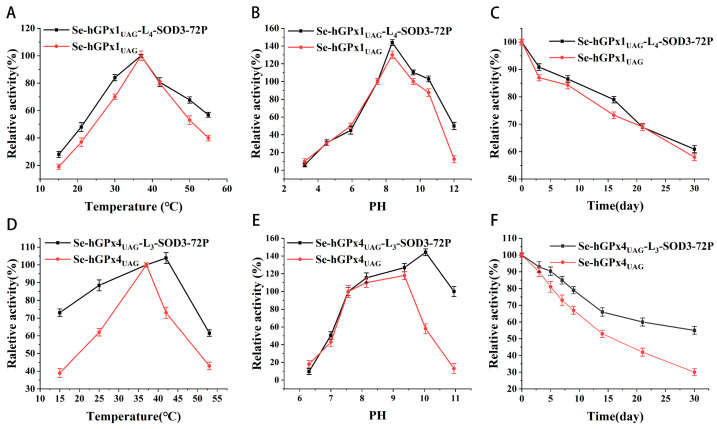
Properties of *Se-hGPx1_UAG_-L_4_-SOD3-72P* and *Se-hGPx4UAG-L3-SOD3-72P*. (**A**,**D**) Relative activity versus temperature. (**B**,**E**) Relative activity versus pH. The GPx activity was determined when the concentrations of GSH and tBuOOH were 1 and 0.3 mM, respectively. The activity at 37 °C and pH 7.4 was set as 100%. (**C**,**F**) Effect of storage time on activity of GPx, and the initial activity at 0 days was set as 100%. Data represent the mean ± SD (*n* = 3).

The GPx activity was measured over a temperature range from 15 to 45 °C and a pH range from 3 to 11. As shown in Figure 2A,B, high values of GPx activity of *Se-hGPx1_UAG_-L_4_-SOD3-72P* were detected between 35 and 40 °C at pH 7.4, with the maximum activity at ~37 °C and the optimal pH was found to be ~8.5, which showed no significant difference with *Se-hGPx1_UAG_*. As shown in Figure 2D,E, the optimal temperature and pH of *Se-hGPx4UAG-L3-SOD3-72P* were found to be ~43 °C and ~10.05, respectively, higher than *Se-hGPx4_UAG_*. To investigate whether the formation of fusion proteins changed the effect of storage time on GPx activity, *Se-hGPx1_UAG_-L_4_-SOD3-72P* and *Se-hGPx4UAG-L3-SOD3-72P* were stored at 4 °C for 30 days. Meanwhile, as a control, *Se-hGPx1_UAG_* and *Se-hGPx4_UAG_* were treated under the same conditions. Their activities were measured at a series of time points as before. Results were shown in Figure 2C,F. After 30 days in storage, the GPx activity of fusion proteins remained ~60%.

### 3.4. Steady State Kinetics of Se-hGPx1_UAG_-L_4_-SOD3-72P and Se-hGPx4_UAG_-L_3_-SOD3-72P

Double-reciprocal plots of initial rate versus the reciprocal substrate concentrations produced a family of parallel lines (Figure S2), indicating that *Se-hGPx1_UAG_-L_4_-SOD3-72P* and *Se-hGPx4UAG-L3-SOD3-72P* all followed a ping–pong mechanism, akin to native GPx. The apparent kinetic parameters were calculated from Equation (1) [3,25].

(1) $$\frac{{\left[E\right]}_{0}}{V_{0}}=\frac{1}{k_{+1}^{\prime }[\mathrm{tBuOOH}]}+\frac{1}{k_{+2}^{\prime }\left[GSH\right]}$$

The *k*′_+1_ value of *Se-hGPx1_UAG_-L_4_-SOD3-72P* and *Se-hGPx4UAG-L3-SOD3-72P* is approximately (3.84 ± 0.17) × 10^6^ M^−1^ S^−1^ and (6.89 ± 0.39) × 10^4^ M^−1^ S^−1^, respectively. The *k*′_+2_ value is approximately (4.89 ± 0.56) × 10^5^ M ^−1^ S^−1^ and (1.13 ± 0.20) × 10^4^ M^−1^ S^−1^, respectively.

### 3.5. Determination of the Synergism of Fusion Proteins

#### 3.5.1. Resistance to Inactivation by H_2_O_2_

**Figure 3:**
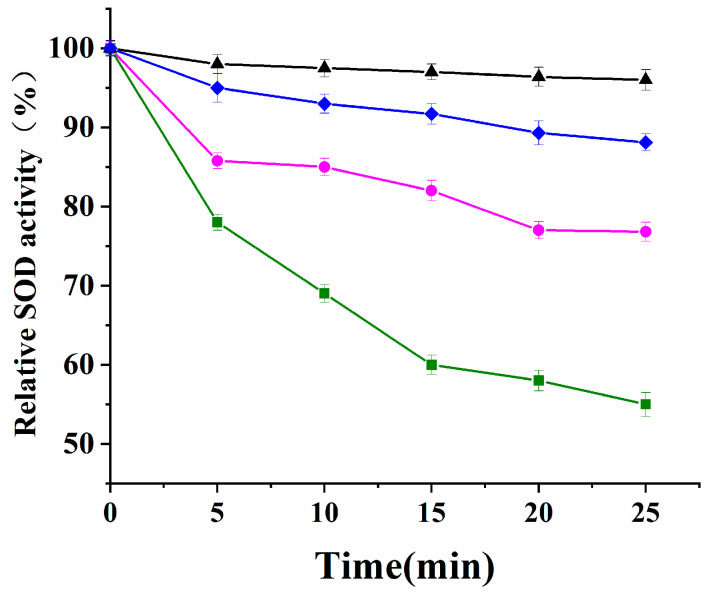
Time course of SOD activity of *Se-hGPx4-L_3_-SOD3-72P* and *Se-hGPx1_UAG_-L_4_-SOD3-72P* incubated with H_2_O_2_. The SOD activity was converted to relative value, and the initial activity at 0 min was defined as 100%. (▲) *Se- hGPx1_UAG_-L_4_-SOD3-72P* was incubated with 1 mM H_2_O_2_ and 2mM GSH in 50 mM Tris-HCl (pH 7.4). (♦) *Se-hGPx4UAG-L3-SOD3-72P* was incubated with 1 mM H_2_O_2_ and 2mM GSH in 50 mM Tris-HCl (pH 7.4). (●) SOD3-72P was incubated with 1 mM H_2_O_2_ and 2mM GSH in 50mM Tris-HCl (pH 7.4). (■) SOD3-72P was incubated with 1 mM H_2_O_2_ in 50mM Tris-HCl (pH 7.4). Data represent the mean ± SD (*n* = 3).

In order to examine the cooperation of *Se-hGPx1_UAG_-L_4_-SOD3-72P* and *Se-hGPx4-L_3_-SOD3-72P*, their resistance to inactivation by H_2_O_2_ was tested using *Se-hGPx1_UAG_-L_4_-SOD3-72P*, *Se-hGPx4-L_3_-SOD3-72P*, and *SOD3-72P*. As shown in Figure 3, the results indicated that the SOD activity of *SOD3-72P* decreased quickly within 25 min of incubation with H_2_O_2_. The addition of 2 mM GSH to the reaction system resulted in a deceleration of SOD activity reduction, although it still experienced a ~33% loss of activity. In contrast, the SOD activity of *Se-hGPx1_UAG_-L_4_-SOD3-72P* with the highest GPx activity remained at about 96% under the same conditions, which was higher than *Se-hGPx4UAG-L3-SOD3-72P* (88%).

#### 3.5.2. *Se-hGPx1_UAG_-L_4_-SOD3-72P* and *Se-hGPx4UAG-L3-SOD3-72P* Aid in Protecting RBCs and the Liver against Oxidative Damage

**Figure 4:**
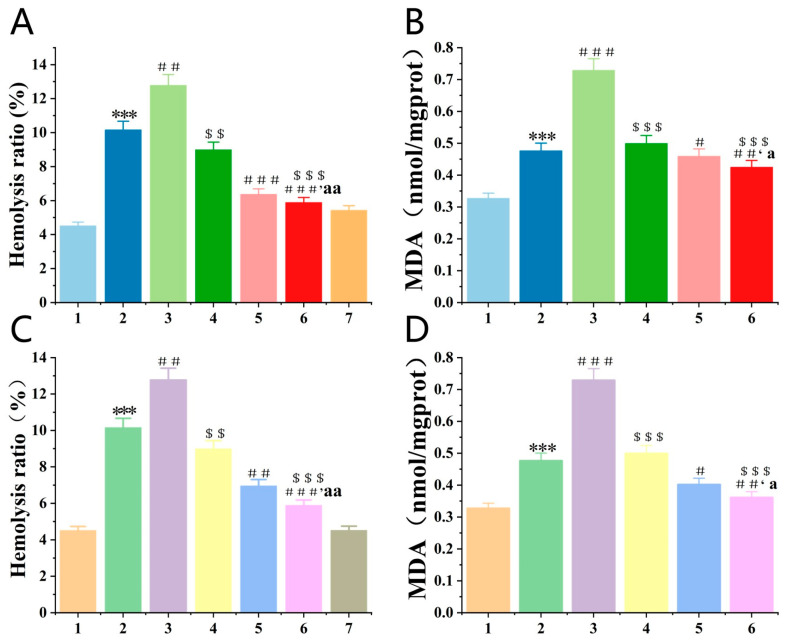
Inhibitory effect of fusion proteins on the hemolysis ratio of RBCs and lipid peroxidation of liver tissue suspension induced by H_2_O_2_. 1: control; 2: damage; 3: damage + *SOD3-72P*; 4: damage + 0.05mM GSH + *SOD3-72P*; 5: damage + 0.05 mM GSH + *Se-hGPx_UAG_*; 6: damage + 0.05mM GSH + fusion proteins; and 7: fusion proteins. (*** *p* < 0.001 vs. control, ^#^ *p* < 0.05, ^##^ *p* < 0.01, ^###^ *p* < 0.001 vs. damage, ^$$^ *p* < 0.01, ^$$$^ *p* < 0.001 vs. damage + *SOD3-72P*, ^a^ *p* < 0.05, ^aa^ *p* < 0.01 vs. damage + 0.05 mM GSH + *Se-hGPx_UAG_*). The hemolysis ratio of RBCs (**A**) and contents of MDA (**B**) in Se-hGPx1_UAG_-L_4_-SOD3-72P. The hemolysis ratio of RBCs (**C**) and contents of MDA (**D**) in *Se-hGPx4UAG-L3-SOD3-72P*. Data represent the mean ± SD (*n* = 3).

Structural changes in RBCs’ membrane lipids, proteins, and hemoglobin (Hb) are known to be caused by excess H_2_O_2_, leading to oxidative hemolysis of these cells [22] and the release of Hb. Thus, measurement of Hb absorbance provides an indication of the degree of RBCs damage. As shown in Figure 4A,C, the hemolysis rate of the H_2_O_2_ groups was greatly higher than the control groups. And all treatment groups could inhibit H_2_O_2_-induced hemolysis in RBCs except the *SOD3-72P* groups. Moreover, the hemolysis ratio of *Se-hGPx1_UAG_-L_4_-SOD3-72P* and *Se-hGPx4UAG-L3-SOD3-72P* fusion protein groups was lower than that of other groups. The toxicity of fusion enzymes was evaluated during the interim period, and there were no significant differences in the hemolysis rates of the fusion enzymes compared to the control groups.

The liver is a vital organ in the human body, playing crucial roles in metabolism and detoxification processes. However, the liver is susceptible to various factors. Drug-induced acute liver failure (ALF) has remained a serious public health issue worldwide over the past few decades. Excessive drugs would cause a series of reactions in the body, producing excess ROS and resulting in oxidative damage to the liver. Malondialdehyde (MDA) can reflect the degree of lipid peroxidation, and it is a representative antioxidant indicator. As shown in Figure 4B,D, the MDA level of damage groups was greatly higher than the control groups, and all treatment groups diminished compared with damage groups except the *SOD3-72P* groups. Moreover, the MDA level in fusion protein groups was lower than those observed in the Se-hGPx_UAG_ and *SOD3-72P* groups.

#### 3.5.3. Inhibitory Effect of *Se-hGPx1_UAG_-L_4_-SOD3-72P* and *Se-hGPx4UAG-L3-SOD3-72P* on Lipid Peroxidation of Yolk Lipoprotein

**Figure 5:**
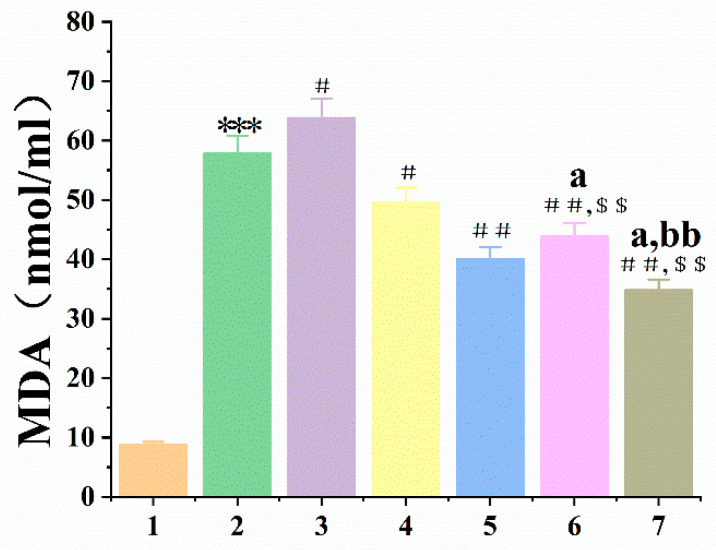
Inhibitory effects of *Se-hGPx4UAG-L3-SOD3-72P* and *Se-hGPx1_UAG_-L_4_-SOD3-72P* on lipid peroxidation induced by FeSO_4_ in vitro. 1: control; 2: damage; 3: damage + *SOD3-72P*; 4: damage + *Se-hGPx1_UAG_*; 5: damage + *Se-hGPx4_UAG_*; 6: damage + *Se-hGPx1_UAG_-L_4_-SOD3-72P*; and 7: damage + *Se-hGPx4UAG-L3-SOD3-72P*. (*** *p* < 0.001 vs. control, ^#^ *p* < 0.05, ^##^ *p* < 0.01 vs. damage, ^$$^ *p* < 0.01 vs. damage + *SOD3-72P*, ^a^ *p* < 0.05 vs. damage + Se-hGPx_UAG_, ^bb^ *p* < 0.01 vs. damage + *Se-hGPx1_UAG_-L_4_-SOD3-72P*). Data represent the mean ± SD (*n* = 3).

Egg yolk is abundant in lecithin, which is easily oxidized by Fe^2+^. In order to compare the protective effect of *Se-hGPx1_UAG_-L_4_-SOD3-72P* with *Se-hGPx4UAG-L3-SOD3-72P* on phospholipid peroxidation damage, a lipoprotein peroxidation damage model was established with FeSO_4_ as an inducer. The results showed that the MDA level of the damage group was greatly higher than the control group, and all treatment groups diminished compared with the damage groups except the *SOD3-72P* groups, while *Se-hGPx4UAG-L3-SOD3-72P* was the lowest (Figure 5).

### 3.6. Exploration of Cys That May Participate in the Formation of Disulfide Bonds

**Figure 6:**
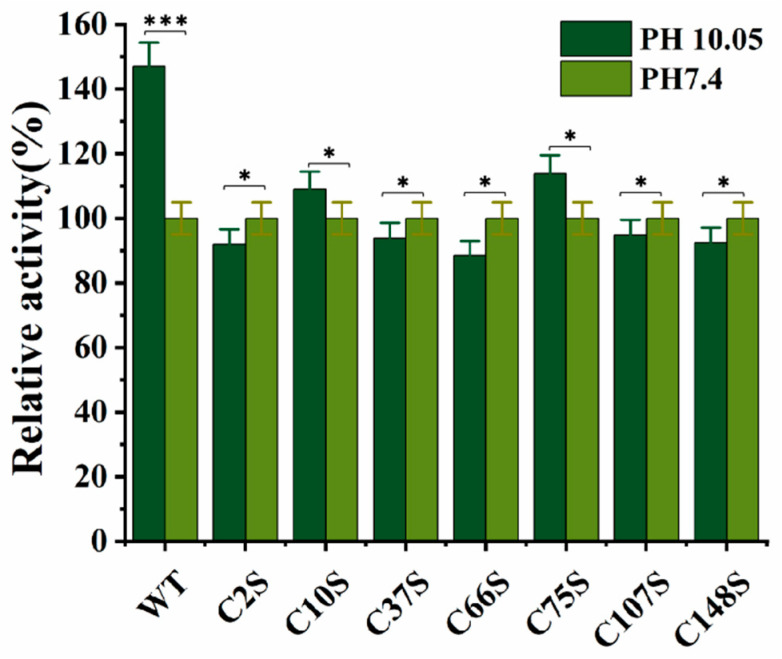
Enzymatic activity of *Se-hGPx4UAG-L3-SOD3-72P*(WT) and mutants at different pH. The activity at pH 7.4 was set as 100%. *** *p* < 0.001, * *p* < 0.05 vs. pH 7.4. Data represent the mean ± SD (*n* = 3).

In order to explore whether the formation of disulfide bonds enhanced the stability of the *Se-hGPx4UAG-L3-SOD3-72P* fusion protein, we expressed several *Se-hGPx4UAG-L3-SOD3-72P* mutants in which seven Cys were replaced with Ser, respectively. According to Table S3, the detected free sulfhydryl groups were all lower than the theoretical values in *Se-hGPx4UAG-L3-SOD3-72P* and mutants, indicating the presence of disulfide bonds. Then, the GPx activity was measured at pH 7.4 and pH 10.05. As shown in Figure 6, the activity of mutants *Se-hGPx4UAG-L3-SOD3-72P*-C2/37/66/107/148S decreased at pH 10.05 compared with that at pH 7.4, similar to that of Se-hGPx4_UAG_. However, mutants of *Se-hGPx4UAG-L3-SOD3-72P*-C10/75S showed the opposite result.

## 4. Discussion

Excessive ROS is often associated with some chronic and degenerative diseases, involving cancer, arthritis, and autoimmune diseases [26,27]. Therefore, it is crucial to maintain the balance between the production and removal of ROS. GPx and SOD play an important role in this process. However, in order to maintain redox balance in vivo, antioxidant enzymes collaborate rather than function independently. The expression of bifunctional enzymes is the basis for studying the synergistic effect of enzymes and obtaining more effective therapeutic drugs.

The previous experimental results showed that the insertion of a linker seems to effectively reduce the adverse consequences caused by the interaction between different domains when constructing bifunctional proteins [18]. In this study, in addition to the most commonly used flexible linker (GGGGS)_3_, in order to further reduce the mutual influence between the two parts of fusion proteins, we also used the long linker S(GGGGS)_4_ to construct the bifunctional enzymes [28].

Among the eight members of GPx, GPx1 is not only widely distributed and high in content but also has the strongest activity, which means that it has a major antioxidant function in the body. Thus, in this study, we first integrated the natural GPx1 gene with the active domain of SOD3 (*SOD3-72P*) to obtain bifunctional proteins using the UTuT6 expression system.

**Figure 7:**
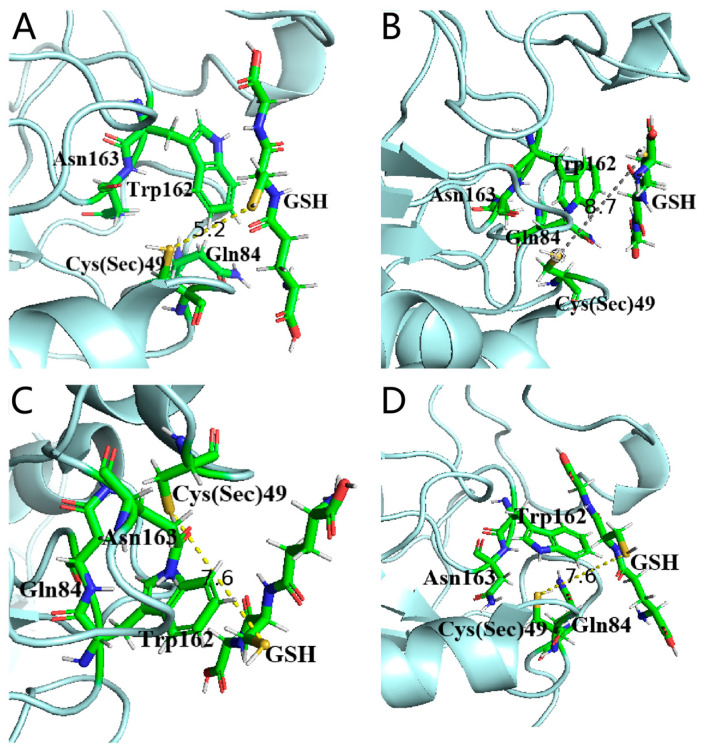
Comparison of putative structures of the GPx active site of natural hGPx1 and fusion proteins predicted using Phyre2. GPx Active site putative structure of (**A**) *(Se)-hGPx1_UAG_*, (**B**) *(Se)-hGPx1^Ser^-L-ApSOD*, (**C**) *(Se)-hGPx1_UAG_-L_3_-SOD-72P*, and (**D**) *(Se)-hGPx1_UAG_-L_4_-SOD-72P*.

Non-reduced SDS-PAGE indicated that *Se-hGPx1_UAG_-L_3/4_-SOD3-72P* all existed as tetramers in the solution, similar to the natural GPx1. This might be due to the fact that the flexible peptides maintain the distance between the GPx and *SOD3-72P* domains and, to some extent, reduce the impact of the additional protein sequences on the quaternary structure of GPx1 [4]. The obtained fusion proteins were assayed for their activities, and there was almost no difference in GPx activity between the two fusion proteins (*Se-hGPx1_UAG_-L_3_-SOD3-72P*: 190 ± 29 U/mg, *Se-hGPx1_UAG_-L_4_-SOD3-72P*: 199 ± 16 U/mg), which was approximately 64% of the activity of Se-hGPx1_UAG_ (321 ± 31 U/mg). The GPx activity of the new fusion proteins acquired in this work has greatly increased compared to the bifunctional proteins (*Se-hGPx1^Ser^-L-ApSOD*: 52.1 ± 4.8 U/mg, Se-Cu/Zn-65P: 112 U/mg) obtained in earlier studies [14,18]. Higher GPx activity seems to reduce H_2_O_2_ to non-toxic alcohols more rapidly, and avoid excessive H_2_O_2_ accumulation. The effect of structural changes on the catalytic efficiency of fusion proteins was further analyzed, and molecular modeling was used to simulate the fusion protein’s structure. In the catalytic cycle of SecGPxs, the selenol reacts with H_2_O_2_ to form selenenic acid, which is reduced by two GSH, forming GSSG and H_2_O [25]. Apart from the catalytic centers including Sec, Gln, Trp, and Asn, the GSH binding site is considered pivotal in eliminating peroxides, and the distance between the Se atom of reactive Sec and the S atom of GSH is believed to be a key factor for its catalytic efficiency. GSH was docked within predicted fusion proteins and native hGPx1 using Edock (Sec at the active site of GPx1 was mutated into Cys) [21]. As shown in Figure 7, the distance between the Cys (Sec) and GSH in *Se-hGPx1^Ser^-L-ApSOD* is 8.7 Å, and the distance is shortened to 7.6 Å in (*Se)-hGPx1_UAG_-L_3_/L_4_-SOD3-72P* with higher GPx activity expressed in the UTuT6 expression system. It could be inferred that the replacement of Cys with Ser in fusion proteins [18] could extend the distance between Se and S atoms, thereby decreasing the activity.

**Figure 8:**
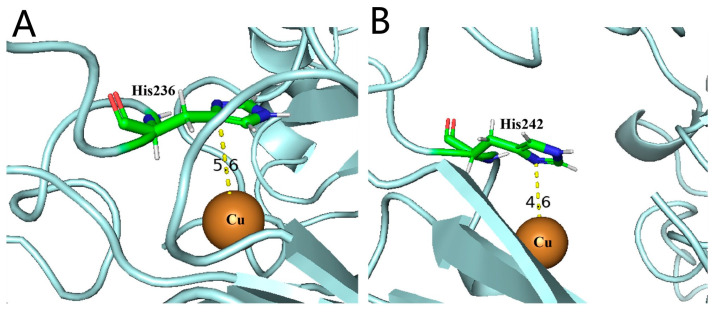
Comparison of putative structures of the SOD active site of fusion proteins predicted using Phyre2. (**A**) SOD active site of *(Se)-hGPx1_UAG_-L_3_-SOD3-72P*, and (**B**) *(Se)-hGPx1_UAG_-L_4_-SOD3-72P*.

After analyzing the structure of SOD3 [13], it was found that SOD3-His113 (*SOD3-72P*-His18) was connected to copper and zinc ions, and the Cu-His113-Zn bridge in SOD3 was intact for the Cu (II) state, while the Cu-His113 bond was broken upon reduction to Cu (I). Copper is the essential metal for the catalytic activity of SOD3, leading to speculation that His18 might be the key amino acid for the activity of *SOD3-72P* [14]. Perhaps the distance between His18 and the copper ion was important for the catalytic activity of proteins. As shown in Figure 8, the Cu ion in *(Se)-hGPx1_UAG_-L_4_-SOD3-72P* with higher SOD activity is closer to His, indicating that the extension of the linker is more conducive to maintaining the correct folding of the SOD active domain.

Then, we studied the enzyme properties of *Se-hGPx1_UAG_-L_4_-SOD3-72P*, and the results showed that its optimal temperature was around 37 °C, implying that the fusion protein could have an anti-oxidative effect normally in the human body. At the same time, we found that although the activity decreased over time, the protein still had about 60% GPx activity after storage for 30 days, indicating that the fusion protein has good stability. In order to further improve the stability of the proteins, some modifications may be made in subsequent studies, such as PEGylation [29]. Analysis of steady state kinetics suggested that the fusion protein followed a ping–pong mechanism in analogy with Se-hGPx1_UAG_. In Equation (1), *k*′_+1_ and *k*′_+2_ are the rate constants for the reaction of the enzyme with tBuOOH and GSH, respectively. The rate constants *k*′_+1_ and *k*′_+2_ of *Se-hGPx1_UAG_-L_4_-SOD3-72P* were (3.84 ± 0.17) × 10^6^ M^−1^ S^−1^ and (4.89 ± 0.56) × 10^5^ M^−1^ S^−1^, respectively, and they were in the same order of magnitude as those of Se-hGPx1_UAG_ produced with the same methods [10], indicating that *Se-hGPx1_UAG_-L_4_-SOD3-72P* had a strong affinity for both tBuOOH and GSH, which was improved compared with an earlier bifunctional enzyme [18].

Furthermore, the results of the synergism experiment revealed that non-enzymatic reduction of H_2_O_2_ was not enough. The GPx activity of the fusion protein effectively inhibited the inactivation of the SOD domain by accelerating the reduction of H_2_O_2_. Though the GPx and SOD activities of *Se-hGPx1_UAG_-L_4_-SOD3-72P* were smaller than those of natural ones, they still played an important role in balancing the harmful effects of ROS.

GPx4 is the only selenoprotein with the ability to reduce phospholipid hydroperoxide, which has been shown to be closely associated with iron death. Based on the unique role of GPx4, in this study, GPx4 and *SOD3-72P* were also connected, yielding two fusion proteins. SDS-PAGE showed that the quaternary structure of the fusion proteins was not changed by *SOD3-72P*, and that they still existed as monomers like natural GPx4.

**Figure 9:**
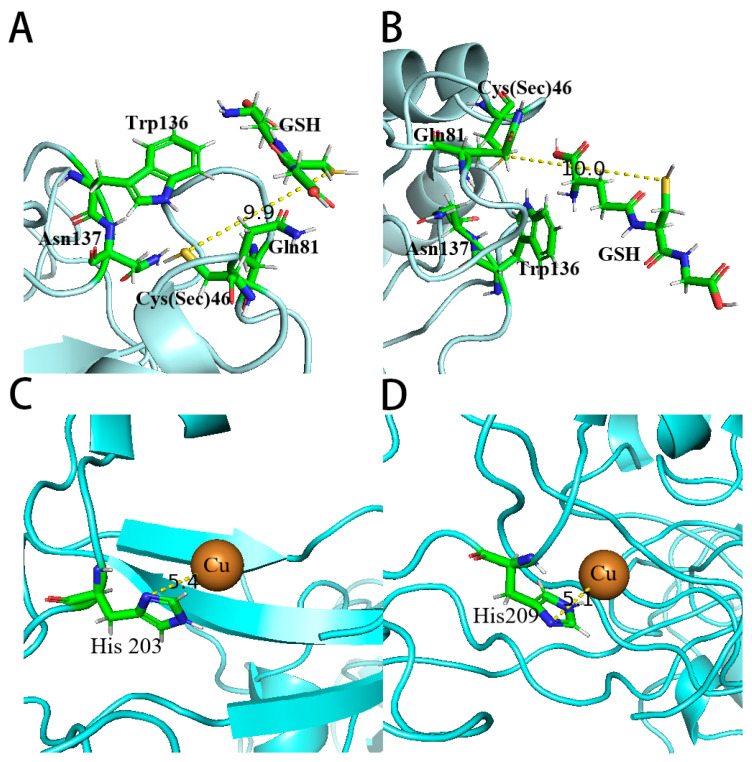
Comparison of putative structures of the GPx active site and the SOD active site of fusion proteins predicted using Phyre2. (**A**) GPx active site structure of putative *(Se)-hGPx4_UAG_-L_3_-SOD3-72P* and (**B**) (Se)-hGPx4_UAG_-L_4_-SOD3-72P. (**C**) SOD active site structure of putative *(Se)-hGPx4_UAG_-L_3_-SOD3-72P* and (**D**) (Se)-hGPx4_UAG_-L_4_-SOD3-72P.

Different from the fusion proteins of GPx1, results showed that there was almost no difference in the activities of GPx and SOD between *Se-hGPx4UAG-L3-SOD3-72P* and *Se-hGPx4_UAG_-L_4_-SOD3-72P*. As shown in Figure 9, the simulated structures of fusion proteins were analyzed, and the distances of the Cys (Sec) and GSH were almost the same. In addition, the distances between the active sites Cu and His in the two fusion proteins were similar, which might be one of the reasons why there was no difference in the activities of the two fusion proteins. Thus, a longer linker does not always improve the activities of fusion proteins. And it is important to select or rationally design a linker to join different fusion protein domains [30].

The results showed that the optimal temperature and pH of *Se-hGPx4UAG-L3-SOD3-72P* for GPx activity were ~43 °C and ~10.05, respectively, higher than *Se-hGPx4_UAG_* (Figure 2D,E). Only 40% of the GPx activity of Se-hGPx4_UAG_ remained, while the remaining activity of *Se-hGPx4UAG-L3-SOD3-72P* protein was over 60%, when the temperature rose to 53 °C. Meanwhile, the residual activity of *Se-hGPx4UAG-L3-SOD3-72P* was about six times higher than that of *Se-hGPx4_UAG_* at pH 11. *Se-hGPx4_UAG_* and *Se-hGPx4UAG-L3-SOD3-72P* were placed under the same conditions to explore the stability of enzymes, and several time points were selected to detect their GPx activity. On the 30th day, the residual activity of *Se-hGPx4UAG-L3-SOD3-72P* was about 55%, while *Se-hGPx4_UAG_* was only about 30%. The above experimental results indicate that the connection of SOD3-72P with GPx4 by utilizing linkers not only changes the optimal temperature and pH of GPx but also increases its alkali and high temperature resistance and improves its stability. These results are not observed in the *Se-hGPx1_UAG_-L_4_-SOD3-72P* fusion protein. Studies have shown that disulfide bonds may stabilize proteins [31,32], and results of Table S3 and Figure 6 indicating that Cys2, Cys37, Cys66, Cys107, and Cys148 residues may participate in the formation of disulfide bonds and enhance the ability of the *Se-hGPx4UAG-L3-SOD3-72P* fusion protein to resist environmental changes. It is undeniable that the formation of fusion proteins would lead to a change in protein conformation.

Selecting a suitable in vitro evaluation method can quickly evaluate the antioxidant capacity of a substance. In this study, we used H_2_O_2_ as an inducer to establish RBCs oxidative hemolysis and liver peroxide damage models, then evaluated the antioxidant capacity of fusion proteins with *Se-hGPx_UAG_* and *SOD3-72P* as controls. The hemolysis rate is a direct indicator of RBCs damage, and the antioxidant capacity of samples can be reflected by measuring the absorbance of HB in each experimental group. As an end product of lipid peroxidation, MDA content can reflect the degree of lipid peroxidation in cells. Figure 5 and Figure 6 showed that *SOD3-72P* not only failed to reduce the hemolysis rate of RBCs and the MDA content, which was produced by liver peroxidation, but also significantly increased it compared with the model groups. SOD catalyzes the dismutation of O_2_^·−^ to O_2_ and H_2_O_2_, and once H_2_O_2_ is not reduced in time, it will lead to the oxidation inactivation of *SOD3-72P* and generate more toxic ·OH, thus causing more serious damage, which is the reason why the hemolysis rate and the MDA content increased in *SOD3-72P* groups. Although the addition of GSH reduced the hemolysis rate and MDA content, the effect was not as obvious as that of fusion proteins with GPx and SOD activities. GPx reduces H_2_O_2_ more efficiently compared with GSH. Therefore, the results indicate that the fusion proteins with GPx and SOD activities have a good synergistic antioxidant capacity. In recent years, researchers have found that RBCs can be used as regulatory factors of cardiovascular function [33], and we believe that the protective effect of *Se-hGPx4UAG-L3-SOD3-72P* and *Se-hGPx1_UAG_-L_4_-SOD3-72P* on erythrocytes would lay a good foundation for subsequent drug development in cardiovascular diseases.

Unlike other members of the GPx family, GPx4 is associated with cell membranes [34], because it is the only selenoprotein that can reduce phospholipid hydroperoxides. When carbon-centered radicals are formed on lipids, they react with oxygen to form lipid peroxyl radicals (LOO · and, subsequently, while forming lipid hydroperoxides (LOOH), initiate a new radical-mediated oxidative chain reaction. When LOOH is a phospholipid hydroperoxide (PLOOH), which could be reduced to the corresponding alcohol by GPx4, it has been shown to initiate a new LPO chain reaction in the absence of GPx4, especially in the presence of Fe^2+^ [35], indicating that GPx4 plays an important role in the termination of phospholipid peroxidation damage. We have demonstrated that *Se-hGPx4UAG-L3-SOD3-72P* and *Se-hGPx1_UAG_-L_4_-SOD3-72P* bifunctional proteins have a good synergistic effect. To substantiate the claim that *Se-hGPx4UAG-L3-SOD3-72P* also has a robust scavenging effect against phospholipid hydroperoxide, we used FeSO_4_ as the inducer to induce lipid peroxidation damage in egg yolk and the individual SOD3-72P, *Se-hGPx_UAG_*, and *Se-hGPx1_UAG_-L_4_-SOD3-72P* groups as controls. MDA content in the FeSO_4_ group was significantly increased compared with the negative control group. The level of MDA in the sample protection groups decreased, which was close to that of the negative control group except for the *SOD3-72P* group, and synergistic antioxidant capacity made the MDA content of fusion protein groups lower than that of individual protein groups. Moreover, the lowest MDA content of *Se-hGPx4UAG-L3-SOD3-72P* indicated that it had a stronger protective effect on lipoprotein peroxidation damage compared with *Se-hGPx1_UAG_-L_4_-SOD3-72P*. The reason is that lecithin is abundant in egg yolks, and phospholipid peroxides are substrates for GPx4. The experimental results show that fusion protein with GPx4 and SOD activities has a good ability to scavenge phospholipid peroxide, which is closely related to the iron death process. This indicates that the *Se-hGPx4UAG-L3-SOD3-72P* protein constructed in this study will also have a good protective effect during the process of iron death in cells.

## 5. Conclusions

In this study, we successfully used the UTuT6 expression system to produce a number of fusion proteins with GPx1/4 and SOD activity. The results showed that *Se-hGPx1_UAG_-L_4_-SOD3-72P* exhibited the highest GPx activity compared with the bifunctional enzymes developed previously and could better protect the active site of SOD; the structural changes in *Se-hGPx4UAG-L3-SOD3-72P* enhanced the stability of the protein; and it has a stronger protective effect on lipoprotein peroxidation damage. *Se-hGPx1_UAG_-L_4_-SOD3-72P* and *Se-hGPx4UAG-L3-SOD3-72P* fusion proteins have good synergistic effects and antioxidant capacities. The successful preparation of bifunctional proteins has far-reaching significance for their clinical application and drug development.

## Figures and Tables

**Table 1:** Activities of fusion proteins.

Catalyst	GPx Activity (U/mg)	SOD Activity (U/mg)
*Se-hGPx1_UAG_*	321 ± 31	ND
*Se-hGPx4_UAG_*	38 ± 2	ND
*SOD3-72P* *Se-Cu/Zn-65P* [14,24]	ND 112	2411.5 ± 113 1254
*Se-hGPx1^Ser^-L-APSOD* [18]	52.1 ± 4.8	101 ± 13
*Se-hGPx1_UAG_-L_3_-SOD3-72P*	190 ± 29	1537 ± 211
*Se-hGPx1_UAG_-L_4_-SOD3-72P*	199 ± 16	2790 ± 125
*Se-hGPx4UAG-L3-SOD3-72P*	20.78 ± 3.1	2506.3 ± 223
*Se-hGPx4UAG-L4-SOD3-72P*	19.2 ± 2.9	2403.6 ± 145

ND, not detected. Data represent the mean ± SD (*n* = 3).

## Data Availability

Data is contained within the article and Supplementary Information.

## Supplementary Materials

The following supporting information can be downloaded at: https://www.mdpi.com/article/10.3390/antiox12091766/s1, Figure S1: SDS-PAGE analysis of *SOD3-72P* protein. M: Marker; 1: reduced SOD3-72P;2: non-reduced SOD3-72P; Figure S2: (A,C) [E]_0_/V_0_ versus 1/[tBuOOH] (mM^−1^) at [GSH] = 1 mM (square), 2 mM (circle), 3 mM(triangle), 5 mM (down triangle). (B,D) [E]_0_/V_0_ versus 1/[GSH] (mM^−1^) at [tBuOOH] = 50 μM (square), 100 μM (circle), 200 μM (triangle), 300 μM (down triangle); Table S1: Primer sequences used for *Se-hGPx_UAG_* fusion proteins cloning; Table S2: Primer sequences used for Se-hGPx4_UAG_ fusion protein mutation; Table S3: Thiol content of *Se-hGPx4_UAG_*/*Se-hGPx4UAG-L3-SOD3-72P* and mutants.
