# Supplementary material for: Study on the Properties and Synergistic Antioxidant Effects of Novel Bifunctional Fusion Proteins Expressed Using the UTuT6 System
Source: Antioxidants (Basel). 2023 Sep 14;12(9):1766. doi: 10.3390/antiox12091766 (PMC10526088; doi:10.3390/antiox12091766)
Supplement: Supplementary file 1 [file antioxidants-12-01766-s001.zip › antioxidants-2575241-supplementary.pdf]

Supplementary information

Study on the properties and synergistic antioxidant effects of novel bifunctional fusion proteins expressed using the UTuT6 system

Qi Yan[1]; Jingyan Wei[1,2,3, *]; Junxia Song[1]; Mengna Li[1]; Xin Guan [1, *]; Jian Song[4, *]

[1] College of Pharmaceutical Science, Jilin University, Changchun, 130021, P. R. China.

[2] Key Laboratory for Molecular Enzymology and Engineering of the Ministry of Education, Jilin University, Changchun, 130000, P. R. China.

[3] Institute of Theoretical Chemistry, Jilin University, 130023 Changchun, PR China.

[4] School of microelectronics, Shanghai University, Shanghai, 201800, China

***The corresponding authors:**

E-mail address: jsong@shu.edu.cn (J. S); weijy@jlu.edu.cn (J. W); guan@jlu.edu.cn(X. G).

Supplementary Results

1. Supplementary tables

Table S1: Primer sequences used for Se-hGPx$_{UAG}$ fusion proteins cloning.

Primers	*Squences 5'→3'*
hGPx1-BamHI-F	CGCGGATCCGATGTGTGCTGCTC
hGPx1$_{UAG}$-linker-R	GAGCCACCTCCGCCTGAACCGCCTCCACCGGCACAGCT GGGCCCTTG
hGPx1$_{UAG}$-long linker-R	CCGCCTGAACCGCCTCCACCAGATCCACCGCCAC CGGAGGCACAGCTGGGCCCTTG
linker-SOD3-72P-F	CGGAGGTGGCTCAGGCGGTGGCGGCTCACACGTTCA CCAGTTCG
SOD3-72P-HindIII-R	CCCAAGCTTTTAGTCTTCACCAGC
SOD3-72P-BamHI-F	CGGGATCCCACGTTCACCAGTTCG
hGPx4-EcoRI-F	CCGGAATTCGATGTGTGCTGCTC
hGPx4$_{UAG}$-linker-R	GAGCCACCTCCGCCTGAACCGCCTCCACCGAAATAGTGG GGCA
hGPx4$_{UAG}$-long linker-R	CCGCCTGAACCGCCTCCACCAGATCCACCGCCAC CGGAGAAATAGTGGGGCA

Gene accession number used in this study shown below: GPx1: NM_000581; GPx4: NM_001367832; SOD3: NM_003102.

Table S2: Primer sequences used for Se-hGPx4$_{UAG}$ fusion protein mutation.

Primers	*Squences 5'→3'*
GPx4-C2S-Ndel-F	GGGAATTCCATATGTCGGCGTCC
GPx4-C10S-Ndel-F	GGGAATTCCATATGTGCGCGTCCCGGGACGACTGGCGCTCGG CGCGCTCC
GPx4-C37S-F	GGCTTCGTGTCGATCGTCACC
GPx4-C37S-R	GGTGACGATCGACACGAAGCC
GPx4-C66S-F	ACGCTGAGTCGGGTTTGCGG
GPx4-C66S-R	CCGCAAACCCGACTCAGCGT

GPx4-C75S-F	CCTTCCCGTCGAACCAGTT
GPx4-C75S-R	AACTGGTTCGACGGGAAGG
GPx4-C107S-F	CAGCAAGATCTCGGTGAACGGGG
GPx4-C107S-R	CCCCGTTCACCGAGATCTTGCTG
GPx4-C148S-F	CGACAAGAACGGCTCGGTGGTGAAGCGCT
GPx4-C148S-R	AGCGCTTCACCACCGAGCCGTTCTTGTCG

Table S3: Thiol content of Se-hGPx4$_{UAG}$ / Se-hGPx4$_{UAG}$-L$_3$-SOD3-72P and mutants

Cys residues reacting with DTNB	Se-hGPx4$_{UAG}$	Se-hGPx4$_{UAG}$-L$_3$-SOD3-72P	C10S	C37S
Theoretical	7	8	7	7
Detected	7.4±0.2	2.78±0.3	1.8±0.32	2.9±0.29

2. Supplementary Figures

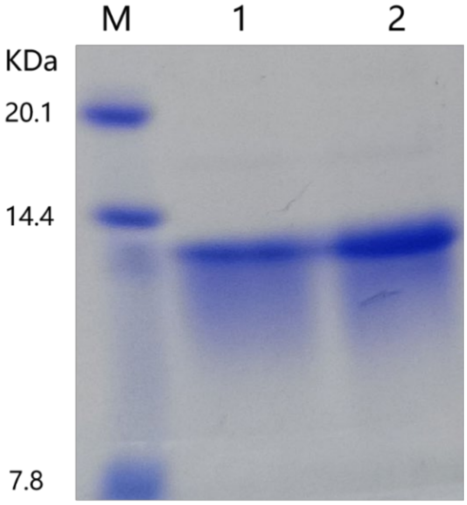

Figure. S1. SDS-PAGE analysis of SOD3-72P protein.

M: Marker; 1: reduced SOD3-72P;2: non-reduced SOD3-72P.

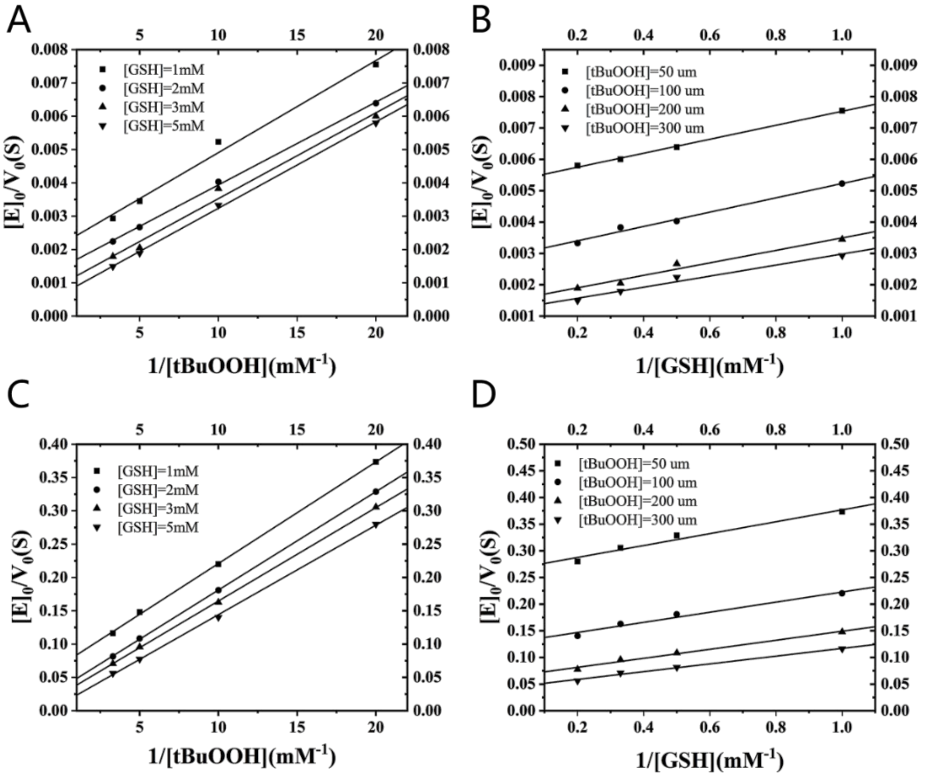

Figure. S2. (A)(C) $[E]_0/V_0$ versus 1/[tBuOOH] (mM^{-1}) at [GSH]=1 mM (square), 2 mM (circle), 3 mM(triangle), 5 mM (down triangle). (B)(D) $[E]_0/V_0$ versus 1/[GSH] (mM^{-1}) at [tBuOOH]=50μM (square), 100 μM (circle), 200 μM (triangle), 300 μM (down triangle).
